# A Brief Mindfulness Intervention for Parents and Children before Pediatric Venipuncture: A Randomized Controlled Trial

**DOI:** 10.3390/children9121869

**Published:** 2022-11-30

**Authors:** Rachel L. Moline, Kaytlin Constantin, Christine T. Chambers, Deborah Powell, Stephen P. Lewis, Laryssa Laurignano, C. Meghan McMurtry

**Affiliations:** 1 Department of Psychology, University of Guelph, Guelph, ON N1G 2W1, Canada; 2 Departments of Psychology and Neuroscience & Pediatrics, Dalhousie University, Halifax, NS B3H 1V7, Canada; 3 Centre for Pediatric Pain Research, IWK Health, Halifax, NS B3K 6R8, Canada; 4 Department of Paediatrics, Schulich School of Medicine & Dentistry, Western University, London, ON N6A 3K7, Canada; 5 Pediatric Chronic Pain Program, McMaster Children’s Hospital, Hamilton, ON L8N 3Z5, Canada

**Keywords:** RCT, mindfulness, children, parents, venipuncture

## Abstract

Background: Routine needle procedures can be distressing for parents and children. Mindfulness interventions may be helpful for parents and children but have not been examined for pediatric needle procedures despite showing benefits in the context of pediatric chronic pain and in lab-based pain tasks. Methods: This preregistered (NCT03941717) two-arm, parallel-group randomized controlled trial examined the effects of a 5 min mindfulness intervention before pediatric venipuncture for parents and children (aged 7–12) compared to a control group on primary outcomes of child pain and fear, secondary outcomes of parent distress, and tertiary outcomes of parent ratings of child pain and fear. Moderators of parent and children’s responses to the intervention were examined: state catastrophizing, trait mindfulness, and experiential avoidance. Results: Sixty-one parent–child dyads were randomized (31 mindfulness; 30 control). Parents and children completed measures, listened to a 5 min audio recording (mindfulness or control), and parents accompanied their child during routine venipuncture. The mindfulness intervention involved breathing and encouraging nonjudgmental attention to one’s experiences, while the control condition involved an unfocused attention task. Three between-subject MANCOVAs assessed for group differences. Child pain and fear rated by children and their parents did not differ between groups. Parents in the mindfulness group were less distressed during the venipuncture than the controls. Parent state catastrophizing may have moderated the intervention effects, such that parents with moderate and high catastrophizing levels had lower distress following the mindfulness intervention versus control. Conclusions: The intervention did not reduce child pain or fear but reduced parent distress. It appeared most helpful for parents catastrophizing about their child’s pain, which is noteworthy as these children are prone to worse outcomes.

## 1. Introduction

Children commonly undergo needles, which can be painful, scary, and distressing for them and their parents [1]. When unmanaged, children’s pain and fear are associated with short- and long-term consequences that negatively impact children, parents, and the healthcare system, including longer procedure times, increased risk of injury, pain, and the need for analgesics during future procedures and healthcare avoidance [2,3]. Mindfulness interventions may help children cope by encouraging adaptive responses to pain stimuli [4]; however, mindfulness interventions have not been examined for children’s needle procedures, despite having shown benefits for children with chronic pain and in coping with short-lasting pain in lab-based studies [5,6]. More specifically, the only two studies examining mindfulness interventions for pediatric acute pain used lab-based cold pressor tasks [5,6]; therefore, further research is needed to understand the potential utility of this intervention for needle-related pain, fear, and distress.

Mindfulness can be defined as the awareness that emerges through paying attention to the present moment and attending to one’s experiences as they unfold with an attitude of openness, curiosity, and nonjudgement [7,8]. Mindfulness is associated with increased wellbeing and has emerged as an effective intervention for myriad concerns, including anxiety and chronic pain [9,10,11,12,13]. Mindfulness interventions also reduce distress during stressful situations [14]. Accordingly, a mindfulness intervention may help parents and children experience less distress during pediatric needles by encouraging them to allow their thoughts, experiences, and feelings to arise and unfold as they are. Mindfulness decouples sensations and automatic evaluations [15]; this is argued to buffer against reflexive reactions such as catastrophizing, suppression, magnification, or experiential avoidance (avoiding and fighting internal experiences), which are prone to exacerbating distress and pain [16]. Indeed, mindfulness appears to facilitate coping with pain through complex mechanisms, including shifts of attention and reactions to pain and related changes in nociception [17,18,19]. A “top-down” shift of attention following mindfulness training is thought to downregulate the nociceptive input of pain at the level of the thalamus, though the exact neurocognitive mechanisms are still unknown [18]. Among individuals with limited or no mindfulness experience, brief mindfulness interventions are thought to engage cognitive control mechanisms, also described as executive functions (e.g., inhibitory control, reappraisal), limiting the incoming nociceptive input [18]. Taken together, mindfulness interventions comprise an emotion-regulatory strategy that can result in pain and distress alleviation [20,21]. There is mounting evidence for the effectiveness of brief mindfulness interventions for facilitating coping and reducing pain in adults experiencing acute pain [22,23]. However, researchers have yet to investigate the potential benefits of a mindfulness intervention for children undergoing a needle procedure.

While distraction (i.e., directing attention away from one’s internal and/or external experiences) continues to be an efficacious pain management intervention for pediatric needles, it is not a panacea [24]. For example, children who view pain as highly threatening are less likely to benefit from distraction as their attention is likely already on the threatening stimuli (e.g., needle); thereby, attempts to direct their attention away are likely less effective [25,26,27,28]. Moreover, all children without congenital insensitivity to pain will experience other pain in their lives [25], thereby emphasizing a need for a repertoire of strategies and interventions that are “matched” to the individual’s needs. Mindfulness may be a helpful solution for when distraction fails, as it allows the individual to maintain their attention on the threatening stimuli and instead provides guidance on how to experience the present moment (hopefully) with less distress.

Although parents play a crucial role in children’s pain experiences, most interventions focus on the child [24]. This leaves the potential of parents as a coping resource untapped and, more concerningly, ignores the potentially deleterious effect that parents’ cognitive-affective experiences may have on children [26]. Troublingly, parents experiencing high distress during children’s experiences with needles are more likely to act in ways that exacerbate children’s pain and fear, and they are less likely to benefit from commonly recommended pain management interventions such as distraction [24,27,28,29,30,31]. Distress reduces parents’ ability to co-regulate their child’s emotions, with parent emotion regulation connecting to how helpful their behaviors are in supporting children’s coping during pain [26,32]. Parents’ distress has been overlooked and under-targeted during painful procedures involving children. A mindfulness intervention focusing on parent emotions may therefore reduce their distress during child venipunctures, thereby increasing parents’ ability to support their child [33]. Mindfulness has been associated with increasing an adult’s attunement to others in the social environment [12], although there has not been a mindfulness intervention for parents as pain observers to date. Taken together, mindfulness may offer an ideal solution to both children’s needle experiences and in guiding helpful parent responses to their child during procedural pain.

This study is the first to (1) examine a mindfulness intervention to support children undergoing pediatric needles and (2) offer a parent-focused mindfulness intervention during pediatric acute pain. This preregistered randomized controlled trial examined the effectiveness of a 5 min parent and child mindfulness intervention before child venipuncture (protocol paper: [33]). It was hypothesized that the mindfulness intervention would reduce child pain, reduce child fear, and reduce parent distress, all as rated by both children and parents. As pain interventions are not “one size fits all”, potential moderators of child and parent responses to the intervention were explored: parent and child state catastrophizing, experiential avoidance, and trait mindfulness [32,34]. Based on the findings that parent and child state catastrophizing relate to worse pain-related experiences [27] and experiential avoidance associated with increased distress in adults [35], it was hypothesized that those experiencing high catastrophizing and high experiential avoidance would benefit more from the mindfulness intervention. In line with past research [6], those with high trait mindfulness were expected to benefit more from the mindfulness intervention.

## 2. Materials and Methods

A detailed study protocol for this trial has been published [33]. This study was a two-arm, blinded, parallel-group, controlled trial, preregistered with Clinical Trials (NCT03941717). Ethics clearance was obtained from the Hamilton Integrated Research Ethics Board at McMaster Children’s Hospital (project #5481) and the Research Ethics Board at the University of Guelph (#19-05-028). The participants were recruited from the outpatient blood-draw lab at McMaster Children’s Hospital, a tertiary care children’s hospital in Ontario, Canada. The inclusion criteria were (1) children between the ages of 7 and 12, (2) undergoing a venipuncture for clinical purposes, (3) being accompanied by a primary caregiver, (4) having proficiency in English sufficient to understand the interventions and complete study measures. Children arriving at the blood-draw lab who appeared to be around 7–12 years of age and their parents were approached for possible recruitment. The children underwent venipunctures for a variety of clinical reasons and were included if accompanied by a primary caregiver. Once enrolled, dyads were randomly assigned to either a mindfulness intervention or the control group. Data were collected between 21 October 2019, and 13 March 2020.

### 2.1. Randomization and Blinding

The parent–child dyads were randomized in a 1:1 ratio to one of the two groups using predetermined blocks of 2, 4, and 6. To generate the randomization schedule, CMM (senior author), who was not directly involved with trial execution, used an electronic researcher randomization tool. Sequentially numbered opaque sealed envelopes (SNOSE; [36] were created by an independent research assistant who was otherwise not involved in the study. The participants were blinded to their group assignment and were not aware of the specific study aims; the participants were the sole outcome raters. It is possible that the participants in the intervention group may have recognized the listening activity as a mindfulness activity. Nurses completing the venipuncture were blinded to group allocation. Three researchers collected the data. One researcher (KC) was aware of group allocation as she opened the SNOSE to learn which listening activity to play for participants; KC was not involved with this study’s data analysis to avoid potential sources of bias. During data collection, RM (first author) and MK (non-author research student) were blinded to group allocation. The participants in each group went through the same procedures, with the one difference being the interventlion within the listening activity, which were the same length, making group assignment indistinguishable for RM and MK during data collection.

### 2.2. Procedure

After obtaining informed consent and assent, the participants completed baseline measures on a tablet. Next, parents and children listened to a five-minute audio recording corresponding with the mindfulness intervention or control condition. Children then underwent a venipuncture with their parent in the room. No efforts were made to change the venipuncture procedures, which were performed as per routine clinical care. The venipuncture characteristics (e.g., use of topical anesthetics) were recorded. Within 1–2 min of the venipuncture, parents and children reported the outcome variables and remaining measures on a tablet.

### 2.3. Interventions

The participants listened to either a mindfulness exercise or an unfocused attention task using over-the-ear, noise-canceling headphones attached to a tablet. Parent and child versions were created for the mindfulness and control conditions, respectively. All four pre-recorded audio files lasted 5 min and were narrated by the same person (RM; first author).

Experimental: Mindfulness-based condition. The participants in this condition listened to a five-minute mindfulness exercise created by Siegel and Bryson [37] modified for the venipuncture context. The exercise began with instructions to mindfully inhale and exhale and bring awareness to one’s thoughts and feelings about the upcoming needle. Parents and children were asked to notice their thoughts, feelings, and worries without judgment, seeing them as “a cloud in the sky” that is changing and temporary.

Control: Unfocused attention condition. In line with previous RCTs with mindfulness interventions which have used mind-wandering/unfocused attention conditions as a control [38,39], the participants in this condition listened to a five-minute unfocused attention exercise modified for the study’s context. The exercise encouraged parents and children to act as they normally would, and let their minds and thoughts wander as usual and continue to do so during the venipuncture. This control condition was selected to better mirror the intervention condition of an audio recording compared to the previous studies in pediatric acute pain involving a mindfulness intervention during cold pressor pain, which had the participants read magazines [6] or engage in a guided imagery task [5].

### 2.4. Measures

Primary Outcomes: The primary outcomes were children’s self-reported ratings of pain and fear experienced during the needle. Two 11-point Numeric Rating Scales (NRS) were completed within two minutes following the venipuncture [40]. Children were asked, “How much pain did you have during the needle?”. The response options ranged from 0, “no pain”, to 10, “very much pain”. The children were also asked, “How scared were you during the needle?”. The response options ranged from 0, “not scared”, to 10, “very scared.” The NRS has been previously utilized to measure pain intensity and fear in children (7 years and older) and during acutely painful procedures [40].

Secondary Outcomes: The secondary outcomes were children’s and parents’ ratings of parent distress during the needle. Two 11-point Numeric Rating Scales (NRS) (researcher generated) were completed within two minutes following the venipuncture. The children were asked, “Tell us how upset you think your parent or caregiver was during the needle”, and the parents were asked, “How distressed were you during your child’s needle?”. For both, the response options ranged from 0, “not at all”, to 10, “extremely”.

Tertiary Outcomes: The tertiary outcomes were parents’ ratings of child pain and fear during the needle. Two 11-point Numeric Rating Scales (NRS) were completed within two minutes following the venipuncture [40]. The parents were asked, “How much pain do you think your child experienced during the needle?”. The response options ranged from 0, “no pain”, to 10, “very much pain”. The parents were also asked, “How scared do you think your child was during the needle?” The response options ranged from 0, “not scared”, to 10, “very scared”.

State catastrophizing (potential moderator): Parents and children completed a six-item Pain Catastrophizing Scale to measure parent state catastrophizing of their child’s pain (PCS-Parent State, PCS-P), and child state catastrophizing of their pain (PCS-Children State, PCS-C), respectively [41,42,43]. State catastrophizing assesses for the presence of exaggerated negative thinking magnifying the threat associated with the feared stimulus (i.e., child’s venipuncture; [41,42,43]. The response options ranged from 0, “not at all”, to 10, “a lot”. Each total score can range from 0 to 60, with higher scores indicating high state catastrophizing levels. The PCS-P and PCS-C have demonstrated reliable psychometric properties in children experiencing acute pain [40,44]. In this sample, Cronbach’s alphas for the PCS-P and PCS-C were high, namely: α = 0.90 and α = 0.81, respectively. These measures were completed before the intervention and venipuncture.

Trait experiential avoidance (potential moderator): The parents completed the Brief Experiential Avoidance Questionnaire (BEAQ) to measure their levels of experiential avoidance, or tendency to avoid, push away and not remain in contact with internal experiences, including thoughts, feelings, and sensations [45]. Experiential avoidance also connects to attempts to eliminate unwanted private experiences, such as memories, emotions, etc. [45]. The response options ranged from 1, “strongly disagree”, to 6, “strongly agree”. The total score can range from 14 to 84, with higher scores indicating high levels of experiential avoidance. The BEAQ demonstrated acceptable internal consistency in a community sample and in psychiatric outpatient adults [45]. Due to a survey error, item 7 was not displayed to any participant; therefore, the total score of the BEAQ was calculated with 14 items instead of 15. In this sample, Cronbach’s alpha of the BEAQ with 14 items indicated strong reliability, α = 0.86. The BEAQ was completed after the venipuncture.

The children completed the eight-item Avoidance and Fusion Questionnaire for Youth (AFQY-8) to measure psychological inflexibility engendered by experiential avoidance and cognitive fusion [46]. The response options ranged from 0, “not at all true”, to 4, “very true”. The total score can range from 0 to 32. Higher scores are consistent with increased psychological inflexibility brought about by high levels of experiential avoidance and cognitive fusion. The AFQY-8 demonstrated acceptable reliability and validity in children in grades 5–10 [46]. In this sample, the Cronbach’s alpha of the AFQY-8 was acceptable, α = 0.79. The AFQY-8 was completed after the venipuncture.

Trait mindfulness (potential moderator): The parents completed the ten-item Cognitive and Affective Mindfulness Scale Revised (CAMS-R) to measure parents’ daily or trait mindfulness [7]. Trait mindfulness relates to one’s capacity to become aware of and remain in contact with present moment experiences (internal and external) while cultivating an attitude of nonjudgment and acceptance. The response options ranged from 1, “rarely/not at all”, to 4, “almost always”. The total scores can range from 0 to 40, with higher scores reflecting greater mindful qualities. The CAMS-R demonstrated acceptable internal consistency, convergent validity, and discriminant validity in the development paper including healthy adults [7]. In this sample, Cronbach’s alpha of the CAMS-R was high, α = 0.80. The CAMS-R was completed after the venipuncture.

Children completed the ten-item Child and Adolescent Mindfulness Measure (CAMM) to measure their mindfulness skills or trait mindfulness [47]. The response options ranged from 0, “never true”, to 4, “always true”. The total scores can range from 0–40, with higher scores indicating more mindfulness. In the development paper, the CAMM demonstrated acceptable internal consistency and convergent and incremental validity in a sample of healthy children aged ten to seventeen [47]. In this sample, Cronbach’s alpha of the CAMM was acceptable, α = 0.75. The CAMM was completed after the venipuncture.

### 2.5. Statistical Methods

Data preparation and baseline characteristics: Both randomization-based [intention-to-treat (ITT)] and adherence-based [per-protocol (PP)] analyses were planned [33]. As all participants completed the intended intervention and no adverse events prohibited completing study procedures, separate ITT and PP analyses were not needed. Statistical analyses were performed using SPSS (IBM SPSS Statistics, New York) and R software. Descriptive statistics were conducted. Responses on outcomes falling outside of 3 SDs from the mean were winsorized (a way to transform into less extreme values) and included in the analysis; this occurred for four data points identified as outliers: one parent rating of child pain intensity (mindfulness group) and three child ratings of pain intensity (control group).

Frequency and descriptive statistics were used to explore participant demographic information. Independent samples t-tests and chi-squared tests were used to assess for potential differences between the mindfulness and control groups in parent and child characteristics (i.e., gender, age, race/ethnicity, child chronic and/or medical condition, parent history of a chronic pain condition, mindfulness experience, parent education, and marital status) and venipuncture characteristics (i.e., pain management interventions, number of needle pokes/ insertions, restraint).

Trial outcomes: A series of between-subject MANCOVAs were completed to assess for differences between the mindfulness and the control group on primary (child report of pain and fear), secondary (caregiver and child report of caregiver distress), and tertiary outcomes (caregiver report of child pain and fear), controlling for child age and gender.

Moderation analyses: Child age and gender were controlled for in the moderation analyses when stated below. The outliers, as indicated by univariate and multivariate methods (i.e., Z-scores more than 3 SDs from the mean, issues of Mahalanobis distance, Cook’s distance, and leverage), were winsorized, including four data points for the following measures: child experiential avoidance (*n* = 1), parent state catastrophizing (*n* = 1), and parent experiential avoidance (*n* = 2). All data for parents (*n* = 61) and children (*n* = 61) were included in the moderation analyses described below. Moderations were conducted following Hayes’ (2014) recommendations, using regression procedures and the PROCESS macro [33]. Predictor and moderator variables were mean-centered. Demographic data, including child age and gender, were entered in the first step when outlined, followed by predictor variables and interaction terms entered together. Post hoc analyses were conducted using simple slopes to determine if the interaction term predicted the outcome variable. Moderation models were examined graphically by group differences in the outcomes for high (+1 SD), moderate (mean), and low (−1 SD) levels of the moderator variables.

Please see the Supplemental Materials for additional analyses (e.g., exploring RCT outcomes in children who did not receive pain management interventions of topical anesthetic and/or video distraction during their venipuncture, moderation analyses).

## 3. Results

### 3.1. Changes in Response to the COVID-19 Pandemic

The following information is reported in alignment with the published guidelines on reporting clinical studies affected by the pandemic [48]. Following the pandemic restrictions in March 2020, it was no longer possible to conduct the trial at McMaster Children’s Hospital; thus, the trial was closed earlier than anticipated. The collected sample of 61 dyads was powered to detect medium to large effects for the planned MANCOVA analyses (sensitivity power analysis; Effect size f^2^ = 0.17, where a medium effect size f^2^ = 0.15 and a large effect size f^2^ = 0.35). As such, analyses were conducted as planned, including the MANCOVAs, with additional t-tests conducted to explore the outcome variables in response to the reduced sample. In keeping with the protocol, the Bonferroni correction for multiple comparisons was removed in the moderation analyses, given the reduced sample size (see protocol paper [33] for the a priori power analyses). After removing the Bonferroni correction, the sample was powered to detect medium to large effects.

### 3.2. Participant Flow

**Figure 1:**
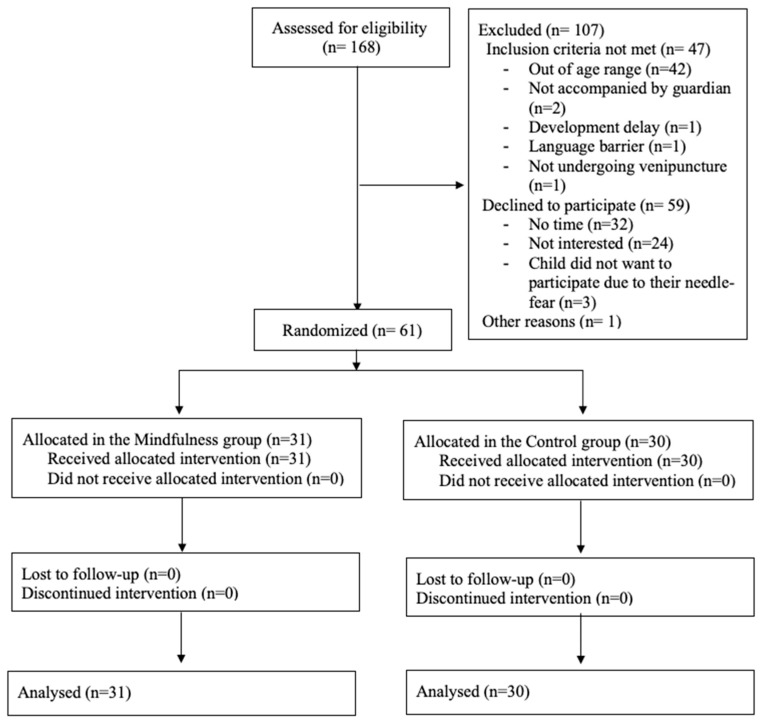
CONSORT Flow Diagram for the trial.

Figure 1 shows the flow chart detailing the participant enrollment, intervention allocation, and data analysis. One-hundred and sixty-eight children and their accompanying parents were approached for participation. Sixty-one parent–child dyads met the eligibility criteria and were randomized. Thirty-one (51%) parent–child dyads participated in the mindfulness condition, and 30 (49%) parent–child dyads participated in the control condition. No adverse events, harms or unintended effects in groups occurred in response to the intervention or during the venipunctures.

### 3.3. Baseline Characteristics

Table 1 shows the characteristics of the participants, including demographic and venipuncture characteristics. Overall, the groups were very similar. Children included both females (~46%) and males (~54%), with ~62% diagnosed with a chronic illness or medical condition (e.g., asthma, juvenile arthritis, diabetes, celiac disease, cystic fibrosis, lupus). Parents included both mothers (~80%) and fathers (~20%), with approximately one-third of parents reporting having a chronic pain condition.

### 3.4. Outcome Variables

Descriptives, primary, secondary, and tertiary outcomes: Table 2 includes the means, standard deviations, and correlations with confidence intervals for the RCT outcomes for the mindfulness and control groups; Supplemental material (Table S1) contains this information for both groups together. Children reported low levels of child pain and fear and parent distress during the venipunctures. Children’s self-reported pain and fear were strongly and positively correlated. Both mindfulness and control groups depicted similar patterns within the correlation matrix, with exceptions involving child ratings of parent distress. Specifically, in the control group, the child self-reports of their pain and fear were positively associated with their perceptions of parent distress. In contrast, within the mindfulness group, there were no significant correlations between child self-report of pain and fear and their perception of parent distress.

Three MANCOVAs were conducted to explore the primary, secondary and tertiary outcomes, controlling for child age and gender. In examining the primary outcomes, child pain and fear did not significantly differ between groups, F (2, 56) = 1.279, *p* > 0.05; Wilks’ lambda = 0.956, partial η^2^ = 0.044 (where η^2^ = 0.01 indicates small effects; η^2^ = 0.06 indicates medium effects; and η^2^ = 0.14 and large effects). The secondary outcomes of parent self-reported distress and child ratings of parent distress did not differ significantly between the groups, F (2, 56) = 2.755, *p* > 0.05; Wilks’ lambda = 0.910, partial η^2^ = 0.090, although the difference was associated with a medium effect size in the hypothesized direction (i.e., parents in the mindfulness condition had lower distress than controls). In exploring the tertiary outcomes of parent perceptions of child pain and fear, no significant differences were found between the groups, F (2, 56) = 0.861, *p* > 0.05; Wilks’ lambda= 0.970, partial η^2^ = 0.030.

Ancillary analyses: Table 3 presents the results of the ancillary t-tests examining between-group differences in the outcome variables. No significant group differences were found in the child self- or parent reports of child pain and fear. However, *t*-tests demonstrated significantly lower parent self- and child-reported parent distress during the venipuncture for parents in the mindfulness condition compared to parents in the control condition.

### 3.5. Moderations

Tables S2 and S3 show the means, standard deviations, and correlations with confidence intervals for child and parent moderator variables and the associated outcomes of parent distress and child pain and fear. The moderation models examining the interaction of the experimental group and child state catastrophizing, experiential avoidance, and mindfulness on child pain or fear were not significant. Parent experiential avoidance and mindfulness also did not emerge as moderators of the intervention’s effect on parent distress. See the Supplemental Materials for the accompanying results write-up.

**Figure 2:**
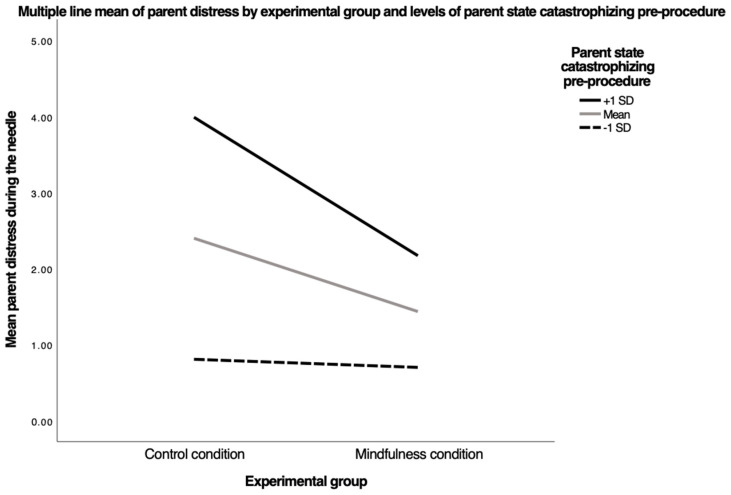
A visual representation of the conditional effects of the experimental group on parent distress during the venipuncture, among those with low (−1 SD), moderate (mean), and high (+1 SD) levels of parent state catastrophizing, pre-venipuncture.

Parent state catastrophizing. The multiple regression model examining parent state catastrophizing prior to the venipuncture and group assignment as predictors of parent procedural distress was significant, F(3, 57) = 13.41, *p* < 0.0001, R^2^ = 0.41. See Figure 2. The experimental group was a significant predictor of parent distress during the venipuncture, b = −0.96, t(57) = −2.12, *p* = 0.03, as was parent state catastrophizing before the venipuncture, b = 0.09, t(57) = 5.24, *p* < 0.001. The interaction of the experimental group and parent state catastrophizing before the venipuncture showed a small effect size but was insignificant, b = −0.10, t(56) = −2.87, *p* = 0.056, indicating that parent state catastrophizing may have moderated the relation between treatment allocation and parent procedural distress. Simple slopes for the association between the groups and procedural distress were tested for −1 SD, mean, and +1 SD parent state catastrophizing levels. For parents low in state catastrophizing pre-venipuncture, there was no relationship between the experimental group and distress during the venipuncture, b = −0.11, t(57) = −0.17, *p* = 0.86. For the average state catastrophizing pre-venipuncture, there was a significant relation between the experimental group and distress during the venipuncture, b = 0.96, t(57) = −2.21, *p* = 0.03. When parents had high state catastrophizing pre-venipuncture, there similarly was a significant relation between the experimental group and distress during the venipuncture b = −1.82, t(57) = −2.93, *p* < 0.005.

### 3.6. Results Summary

No differences were observed between the mindfulness and control conditions on children’s pain and fear, measured by child self-report and parent report. Compared to the control group, parents in the mindfulness group were rated as less distressed by both children and parents. This finding was moderated by parental state catastrophizing such that the parents experiencing moderate and high levels of state catastrophizing experienced less distress following the mindfulness intervention. No other moderators, including parent and child trait mindfulness and experiential avoidance, or child state catastrophizing, were significant.

## 4. Discussion

This is the first study to (1) examine a mindfulness intervention (of any duration) for pediatric needle procedures and (2) offer a parent-focused mindfulness intervention during children’s painful procedures. The 5 min mindfulness intervention did not reduce child pain or fear during their venipunctures; however, it reduced parent distress as rated by parents and their children. Though child fear has not been previously explored in the context of mindfulness interventions, research involving a mindfulness intervention for cold pressor pain among 10- to 18-year-olds similarly found no differences in pain intensity following a brief mindfulness exercise [5,6]. Perhaps this pattern of results is unsurprising, as mindfulness interventions aim to adjust psychological processes, not the sensory experience captured via pain intensity ratings [18,30,49,50,51,52]. As such, this intervention may have affected children’s experiences in ways not captured by the outcome variables (i.e., pain unpleasantness). Further multidimensional assessment of children’s pain experiences is necessary to illuminate the effects of mindfulness interventions [53]. Developmental considerations are critical, as young children primarily characterize their pain in sensory and affective terms, whereas older children are more aware of the cognitive-evaluative aspects of pain experiences [54]. Additionally, in this sample, ~25% of children received an additional pain intervention (e.g., numbing cream), and floor effects of pain and fear were observed. Possibly, in longer or more invasive procedures, a different pattern of results may have emerged. As mindfulness skills develop through practice, the “dose” of the intervention and repeated practice may be particularly relevant for children [8], warranting research involving longer mindfulness interventions (e.g., multiple sessions) to examine the intervention’s effect. It is also possible that a brief mindfulness intervention may not be a suitable intervention for procedural pain in this population. One possible consideration is that individuals use cognitive resources to focus their attention on the present moment. Therefore, during a stressful situation, individuals may be less able to maintain their attention in a particular way and regulate their emotional experience, especially if cultivating an attitude of openness and awareness is new [55]. Therefore, a stronger “dose” of mindfulness training may be required to confer benefits. Future research should determine if, and then subsequently, how much training is required is to benefit youth. Although the duration and frequency of mindfulness interventions have significantly varied across research, many challenges are associated with implementing intensive mindfulness training involving multiple sessions (e.g., patient availability/adherence; resources; trained personnel). Longer interventions may decrease the likelihood of the intervention being translated into routine practice, thereby evidencing the need for establishing a minimally effective dose, which ideally would improve the feasibility and accessibility of such an intervention, particularly during instances of acute pain when pain management interventions are already infrequently offered.

The mindfulness intervention moderately reduced parent procedural distress (self, child ratings), and this was magnified (large effects) in the subsample who did not receive an additional pain management intervention (see Table S4). This is a critical finding as parent distress relates to higher child distress and pain during children’s procedures [26,56,57,58,59,60,61,62]. Theoretically, lower levels of parental distress during children’s painful procedures would be beneficial for themselves and their children [31]. However, further research is needed, as this intervention did not improve child pain and fear despite benefitting parents.

Parent emotion regulation during their child’s pain is critical; parents’ self-oriented distress can limit their child’s ability to co-regulate [26,63]. As this intervention asked parents to acknowledge their worries, this “turning towards” might have lowered their stress, particularly if they are likely to rely on avoidant strategies, which often lead to more distress [64,65,66,67]. Further, parents experiencing high anxiety cope better when implementing emotion regulation strategies that direct their attention toward the feared experience versus directing their attention away [32]. As such, the intervention may have reduced parent distress via attentional deployment, thereby facilitating an emotion regulation strategy “matched” to those experiencing distress. As the mindfulness intervention was delivered before the procedure, it arguably did not require additional self-regulatory resources to adjust their behavior during the venipuncture. Perhaps reducing parent distress beforehand enabled them to be present with their children during the procedure. Indeed, the importance of parents’ active involvement in children’s pain management is essential [68]; in lowering their stress first, efforts to involve them as active agents in children’s coping may increase [69] and may guard against potentially damaging effects of unmanaged distress. Future studies should investigate parental procedural behavior and cognitive-affective experiences following mindfulness interventions.

This is the first study to examine parent and child state catastrophizing, mindfulness, and experiential avoidance as moderators of a mindfulness intervention for pediatric venipuncture. It builds on work examining child catastrophizing and mindfulness as moderators of a mindfulness intervention for experimental pain [6]. Contrary to expectations, child state catastrophizing and parent and child levels of experiential avoidance and trait mindfulness did not moderate the effects of the group on the outcome variables. As with any measure, the Child Pain Catastrophizing Scale has limitations and may not be sensitive to the developmental and cognitive considerations specific to children’s pain experiences and perceptions thereof [70]. The lack of significant findings related to child state catastrophizing is similar to that of Petter and colleagues, who found that child trait catastrophizing did not moderate the impact of the mindfulness group on experimental pain intensity in adolescents [6].

Ancillary analyses showed that the intervention was helpful for parents who initially viewed their children’s impending pain as moderately or highly threatening, which is promising as these parents represent a vulnerable group [27,28,29,71]. Specifically, parents experiencing moderate to high state catastrophizing before the venipuncture who underwent the mindfulness intervention had lower levels of distress experienced during the venipuncture versus the parents in the control group. This is a noteworthy finding, as other research has demonstrated that parent-targeted interventions to support child coping, including parent-led distraction, are less effective for highly distressed and anxious parents [24,72,73]. In contrast to most parent-focused interventions, mindfulness interventions do not ask parents to change their behavior during their child’s pain and, instead, shine a light on parents’ cognitive-affective experiences. This may be a more effective in-road to helping parents experiencing distressing thoughts and feelings as opposed to didactic instructions on how to behave during the procedure.

Future research should explore how fostering children’s mindful awareness of their experiences during procedures relates to their fear, pain, and unpleasantness experienced over time. Focusing on moment-to-moment experiences during venipunctures may be initially more frightening, as indicated by a small, nonsignificant effect of higher child self-report of fear in the mindfulness group. Perhaps with repeated exposure, this “turning towards” the feared experience may yield a different pattern of results, such as the benefits evidenced in youth with chronic pain [74,75]. Although distracting oneself from pain can be efficacious in the short term, it may not translate into an adaptive strategy in the long term and may be ineffective for those who view pain as highly threatening [6,76,77]. Specifically, for those experiencing high catastrophizing or fear, distraction may not be consistently possible or helpful [78,79]. It seems prudent that children who are experiencing catastrophic thoughts and fear relating to their pain be exposed to and offered interventions suited to their needs.

The current study has limitations. The premature closure of the trial in response to COVID-19 restrictions resulted in adjustments to the analytic plan, given the reduced sample. The study setting posed a potential limitation, given the variable noise levels and distractions occurring in the waiting room. To mitigate this issue, the headphones were noise-canceling, and participants were strategically seated and instructed to close their eyes during the listening activity. Another consideration is the differences between the interventions corresponding with each condition. Specifically, the mindfulness intervention script included more words than the control condition script, resulting in more silence and less time talking during the intervention in the control condition. This could have impacted the attention and engagement of participants during the audio recording. Additionally, the perceived helpfulness of the intervention may have played a role, although this was not measured. As well, perhaps there was a positive expectation bias, which may have contributed to the floor effects of pain and fear ratings in the sample overall. Lastly, the timing of the interventions relative to the venipuncture procedure are also important to consider. As mindfulness exercises require cognitive resources, typically top-down processes for novices, there is a risk that participants would be less able to maintain this attention and regulate their emotional experience during the venipuncture. However, it was hypothesized that the mindfulness intervention might reduce emotional distress before the procedure, and self-regulatory resources may be more available during painful procedures for those with little mindfulness experience. Additionally, as discussed by Petter [6], having the intervention prior to the procedure may also reduce the potential analgesic effects of distraction that may have been provided by listening to an audio recording during the needle. In sum, while it is possible that there was a dissipation of the intervention’s effects, the potential confounds of possible distraction and/or an analgesic effect of listening to audio during were removed. Further, listening to an activity during the venipuncture may also have impacted the parental ability to be present with their child, which was a proposed mechanism of action of the intervention.

The strengths of this work include the embedding of a rigorous RCT design within a fast-paced clinical environment, which provides preliminary support for the ecological validity of the findings. The trial was feasible and offered initial evidence for the effectiveness of a brief, inexpensive mindfulness intervention for parents, not requiring trained personnel for implementation or strong mindfulness skills in parents. This clinical sample included children and parents with different health statuses. Parent and child demographic and venipuncture characteristics were largely evenly distributed between groups. Additionally, all participants adhered to the study protocol and assigned conditions, and there were no participant dropouts or missing data for the trial’s outcome variables.

## 5. Conclusions

Needle procedures are common, and, given the frequency of these procedures in childhood, it is troubling that more efforts are not put forth towards helping all children cope with the procedure, and little attention is paid to their associated thoughts and feelings or the experience of parents [2]. Although for some children, it might appear as “just a poke,” early pain experiences are foundational [2,80], meaning that childhood pokes could be an opportunity to empower children and their parents to better handle these potentially distressing situations. This examination of a child and parent-focused mindfulness intervention for children’s painful procedures shows that it reduced parent distress and seemed most helpful for parents catastrophizing about their child’s pain. Although preliminary, these results are important because parents who catastrophize about their child’s pain may not respond to other interventions, are at greater risk of engaging in behaviors that exacerbate their children’s pain, and arguably need more support before their children’s procedures.

Findings indicated that the 5 min mindfulness intervention did not improve children’s pain or fear; this may be related to several factors such as, but not limited to, the sample demographics (e.g., child age and developmental stage, level of mindfulness skills), and the mindfulness intervention used (e.g., breath awareness and mindful imagery approach, duration, and timing of the intervention). Mindfulness training involves meta-cognitive abilities and self-regulation, which are in development throughout childhood and adolescence [81,82]. The current study does not provide evidence for the efficacy of a brief mindfulness intervention for youth in this sample but adds to the literature by demonstrating a “null” finding. Many questions remain unanswered, and future lines of inquiry warrant consideration. For example, it is unclear how mindfulness interventions may impact youth’s pain experiences or confer any benefit for procedural pain. Thus, researchers examining mindfulness interventions for children’s pain are encouraged to examine children’s outcomes, including, but not limited to, pain intensity, fear, and unpleasantness to better capture children’s cognitive and affective experiences. However, more research in developing self-report measures of children’s cognitive-affective dimensions of pain is needed [53]. In addition, as mindfulness interventions aim to target cognitive-affective experiences, developing pain-specific measures of these constructs is crucial to assessing the effectiveness of such interventions [53].

Children’s pain experiences are interwoven within a social environment. The reductions in parents’ distress seen following the mindfulness intervention are theoretically consistent with biopsychosocial approaches to pain management and a promising finding, yet we did not see differences in children’s pain intensity. Future research is encouraged to explore interventions for parental distress during pediatric needle procedures and subsequent investigation as to how this impacts child pain experiences. Given the profound role of parents in children’s socialization, it is critical to teach parents ways to manage their distress during their children’s pain, for both them and their children.

## Figures and Tables

**Table 1:** Baseline demographic characteristics of participants and venipuncture characteristics.

Variable	Mindfulness (*n* = 31) *n* (*%*)	Control (*n* = 30) *n* (%)	Total (*n* = 61) *n* (%)	Significant Difference between Groups?
**Parent characteristics**				
Gender				No; *Χ*^2^(1) ≥ 3.50, *p* = 0.06
Female	22 (71.00)	27 (90.00)	49 (80.32)	
Male	9 (29.00)	3 (10.00)	12 (19.67)	
Age (M_years_ ± SD)	43.00 (6.87)	41.13 (4.26)	42.08 (5.77)	No; *t*(59) = −1.27, *p* = 0.21
Chronic pain condition				No; *Χ*^2^(1) ≥ 4.15, *p* = 0.52
Yes	8 (25.80)	10 (33.30)	18 (29.50)	
No	23(74.20)	20 (66.70)	43 (70.50)	
Past mindfulness experience				No; *Χ*^2^(1) ≥ 4.15, *p* = 0.52
Yes	8 (25.80)	10 (33.30)	18 (29.50)	
No	23 (74.20)	20 (66.70)	43 (70.50)	
Parent race/ethnicity ^a^				
Indigenous	0 (0)	0 (0)	0 (0)	---
White/European	22 (71.0)	23 (76.7)	45 (73.8)	No; *Χ*^2^(1) ≥ 2.56, *p* = 0.61
Black/African	4 (12.9)	0 (0)	4 (6.6)	Yes; *Χ*^2^(1) ≥ 4.14, *p* = 0.04
Southeast Asian	0 (0)	1 (3.3)	1 (1.6)	No; *Χ*^2^(1) ≥ 1.05, *p* = 0.31
Arab	3 (9.7)	2 (6.7)	5 (8.2)	No; *Χ*^2^(1) ≥ 0.18, *p* = 0.67
South Asian	2 (6.5)	0 (0)	2 (3.3)	No; *Χ*^2^(1) ≥ 2.00, *p* = 0.16
Latin American	0 (0)	2 (6.7)	2 (3.3)	No; *Χ*^2^(1) ≥ 2.14, *p* = 0.14
West Asian	0 (0)	1 (3.3)	1(1.6)	No; *Χ*^2^(1) ≥ 1.05, *p* = 0.31
Other	0 (0)	2 (6.7)	2 (3.3)	No; *Χ*^2^(1) ≥ 2.14, *p* = 0.14
**Child characteristics**				
Gender				No; *Χ*^2^(1) ≥ 3.99, *p* = 0.53
Female	13 (41.94)	15 (50)	28 (45.90)	
Male	18 (58.10)	15 (50)	33 (54.10)	
Age (M_years_ ± SD)	9.81 ± 1.60	10.13 ± 1.63	9.95 ± 1.59	No; *t*(59) = 0.789, *p* = 0.433
Chronic illness(es) or medical condition(s)				No; *Χ*^2^(1) ≥ 0.13, *p* = 0.72
Yes	20 (64.50)	18 (60.00)	38 (62.30)	
No	11 (35.50)	12 (40.00)	23 (37.70)	
Past mindfulness experience				No; *Χ*^2^(1) ≥ 0.138, *p* = 0.71
Yes	13 (41.90)	14 (46.70)	27 (44.30)	
No	18 (58.10)	16 (53.30)	34 (55.70)	
Child race/ethnicity				
Indigenous	0 (0)	0 (0)	0 (0)	---
White/European	22 (71.0)	24 (80)	46 (75.4)	No; *Χ*^2^(1) ≥ 0.67, *p* = 0.41
Black/African	4 (12.9)	1 (3.3)	5 (8.2)	No; *Χ*^2^(1) ≥ 1.86, *p* = 0.17
Southeast Asian	2 (6.5)	1 (3.3)	3 (4.9)	No; *Χ*^2^(1) ≥ 0.32, *p* = 0.57
Arab	3 (9.7)	2 (6.7)	5 (8.2)	No; *Χ*^2^(1) ≥ 0.18, *p* = 0.67
South Asian	2 (6.5)	0 (0)	2 (3.3)	No; *Χ*^2^(1) ≥ 2.00, *p* = 0.16
Latin American	0 (0)	2 (6.7)	2 (3.3)	No; *Χ*^2^(1) ≥ 2.14, *p* = 0.14
West Asian	0 (0)	1 (3.3)	1 (1.6)	No; *Χ*^2^(1) ≥ 1.05, *p* = 0.30
Other	1 (3.2)	2 (6.7)	3 (4.9)	No; *Χ*^2^(1) ≥ 0.39, *p* = 0.53
**Venipuncture** **characteristics**				
Pain management intervention				No; *Χ*^2^(1) ≥ 2.44, *p* = 0.12
Yes ^b^	5 (16.10)	10 (33.30)	15 (24.60)	
Numbing spray	4 (12.9)	8 (26.67)	12 (19.67)	
Distraction (movie/video playing)	1 (3.20)	3 (10.00)	4 (6.56)	No (all children had one poke)
No	26 (83.90)	20 (66.70)	46 (75.41)	
Number of needle pokes required for the venipuncture				
1 poke	31 (100.00)	30 (100.00)	61 (100.00)	
2+ pokes	0 (0.00)	0 (0.00)	0 (0.00)	
Additional nurse to hold child’s arm (with assent)	1 (3.2)	2 (6.70)	3 (4.92)	No; *Χ*^2^(1) ≥ 0.39, *p* = 0.53

Note: ^a^ Parent and child race/ethnicity frequencies and percentages total to more than 100% as participants could choose more than one option. ^b^ One child in the control group used both numbing spray and distraction. Distraction, as coded here, involved a movie playing in the room during the venipuncture.

**Table 2:** Means, standard deviations, and correlations with confidence intervals for RCT outcomes for the mindfulness group (top) and control group (bottom).

Variable	*M*	*SD*	1	2	3	4	5	6
1. Child pain	Mind 2.55 Cont 2.57	2.67 2.80		0.84 ** [0.70, 0.92]	−0.13 [−0.46, 0.23]	0.47 ** [0.14, 0.71]	0.37 * [0.02, 0.64]	0.41 * [0.07, 0.67]
2. Child fear	Mind 2.61 Cont 2.03	2.88 2.51	0.78 ** [0.58, 0.89]		0.11 [−0.25, 0.45]	0.43 * [0.09, 0.68]	0.34 [−0.02, 0.62]	0.44 * [0.10, 0.69]
3. Child rating of parent distress	Mind 0.61 Cont 1.37	1.28 2.37	0.43 * [0.08, 0.68]	0.64 ** [0.36, 0.81]		0.26 [−0.10, 0.56]	0.21 [−0.16, 0.52]	0.10 [−0.26, 0.44]
4. Parent distress	Mind 1.39 Cont 2.50	1.45 2.61	0.45 * [0.10, 0.69]	0.62 ** [0.33, 0.80]	0.65 ** [0.38, 0.82]		0.72 ** [0.49, 0.85]	0.69 ** [0.44, 0.84]
5. Parent report of child pain	Mind 2.58 Cont 3.07	2.14 2.21	0.42 * [0.07, 0.68]	0.48 ** [0.15, 0.72]	0.62 ** [0.33, 0.80]	0.71 ** [0.47, 0.85]		0.67 ** [0.41, 0.83]
6. Parent report of child fear	Mind 3.23 Cont 3.87	2.72 2.92	0.56 ** [0.25, 0.77]	0.70 ** [0.45, 0.84]	0.64 ** [0.37, 0.82]	0.76 ** [0.55, 0.88]	0.70 ** [0.46, 0.85]	

Note: *M* and *SD* represent mean and standard deviation, respectively. The outcome variables were all ranked on 11-point (0–10) Likert scales, with 10 corresponding with high levels of the outcome variable. “Mind” and “Cont” represent the mindfulness and control groups, respectively. Values in square brackets indicate the 95% confidence interval for each correlation. * *p* < 0.05. ** *p* < 0.01.

**Table 3:** Means, standard deviations, and *t*-tests comparing differences between groups on the RCT outcome measures.

Outcome Variable	Between Group Differences	Cohen’s *d* (95% CI)
1 Child fear	*p* > 0.05	−0.21 (−0.72,.29)
1 Child pain	*p* > 0.05	0.007 (−0.50, 0.51)
2 Child rating of parent distress	*p* = 0.009 **	0.34 (−0.17, 0.85)
2 Parent state distress	*p* = 0.002 **	0.53 (0.02, 1.04)
3 Parent rating of child fear	*p* > 0.05	0.23 (−0.28, 0.73)
3 Parent rating of child pain	*p* > 0.05	0.27 (−0.24, 0.77)

Note: (*N* = 61 for all; *n* = 31 in the mindfulness group, *n* = 30 in the control group). Cohen’s *d* = 0.2, 0.5 and 0.8 represent a small, medium, and large effect size, respectively. ** *p* < 0.01.

## Data Availability

The datasets (including video recordings and written transcripts) have not been made publicly available due to the risk of the identifying nature of these data for participants.

## Supplementary Materials

The following supporting information can be downloaded at: https://www.mdpi.com/article/10.3390/children9121869/s1, Table S1: Means, standard deviations, and correlations with confidence intervals for the RCT outcome variables including both the mindfulness and control groups, Table S2: Means, standard deviations, and correlations with confidence intervals for child potential moderators and pain and fear including the control and mindfulness groups, Table S3: Means, standard deviations, and correlations with confidence intervals for parent potential moderators and distress including the control and mindfulness groups, Table S4: Means, standard deviations, and t-tests comparing group differences on the RCT outcome measures, excluding 15 children who used numbing spray and/or used distraction of a video playing during the venipuncture.

## Sample Availability

Given the strong reliability observed in this sample, the total score for the scale using the collected 14 items was calculated.
