# Supplementary material for: A Brief Mindfulness Intervention for Parents and Children before Pediatric Venipuncture: A Randomized Controlled Trial
Source: Children (Basel). 2022 Nov 30;9(12):1869. doi: 10.3390/children9121869 (PMC9776747; doi:10.3390/children9121869)
Supplement: Supplementary file 1 [file children-09-01869-s001.zip › children-1983164-supplementary.pdf]

Table S1.

Means, standard deviations, and correlations with confidence intervals for the RCT outcome variables including both the mindfulness and control groups

Variable	*M*	*SD*	1	2	3	4	5
1. Child pain	2.56	2.71					
2. Child fear	2.33	2.70	.80**<br>[.69, .88]				
3. Child rating of parent distress	0.98	1.92	.22<br>[-.03, .45]	.37**<br>[.14, .57]			
4. Parent distress	1.93	2.16	.42**<br>[.19, .61]	.46**<br>[.24, .64]	.58**<br>[.38, .73]		
5. Parent report of child pain	2.82	2.17	.40**<br>[.16, .59]	.39**<br>[.15, .58]	.47**<br>[.24, .64]	.69**<br>[.53, .80]	
6. Parent report of child fear	3.54	2.81	.49**<br>[.27, .66]	.54**<br>[.33, .70]	.46**<br>[.23, .63]	.71**<br>[.56, .82]	.69**<br>[.53, .80]

Note. All outcome variables were rated on a Numerical Rating Scale. *M* and *SD* represent mean and standard deviation, respectively. Values in square brackets indicate the 95% confidence interval for each correlation. $^{**}p < 0.01$

Table S2.

Means, standard deviations, and correlations with confidence intervals for child potential moderators and pain and fear including the control and mindfulness groups

Variable	*M*	*SD*	1	2	3	4
1. Child trait mindfulness	24.03	6.74				
2. Child experiential avoidance	15.97	6.07	-.67** [-.79, -.51]			
3. Child state catastrophizing before procedure	22.92	10.79	-.21 [-.44, .04]	.03 [-.22, .28]		
4. Child pain	2.56	2.71	-.15 [-.38, .11]	.01 [-.24, .26]	.40** [.17, .59]	
5. Child fear	2.33	2.70	-.18 [-.41, .07]	.04 [-.21, .29]	.47** [.24, .64]	.80** [.69, .88]

Note. M and *SD* are used to represent mean and standard deviation, respectively. Values in square brackets indicate the 95% confidence interval for each correlation. ***p* < 0.01.

Table S3.

Means, standard deviations, and correlations with confidence intervals for parent potential moderators and distress including the control and mindfulness groups

Variable	*M*	*SD*	1	2	3
1. Parent trait mindfulness	29.52	4.49			
2. Parent experiential avoidance[a]	44.98	11.19	-.13<br>[-.37, .13]		
3. Parent state catastrophizing before procedure	14.84	13.04	-.10<br>[-.34, .16]	.19<br>[-.06, .43]	
4. Parent self-reported distress during procedure	1.93	2.16	.02<br>[-.23, .27]	.10<br>[-.16, .34]	.54**<br>[33, .69]

Note.[a] Item 7 on the Brief Experiential Avoidance Questionnaire was not displayed to any participant as the question was missing from the survey. Due to the missing item, the total score of the BEAQ was calculated with 14 items instead of 15. *M* and *SD* are used to represent mean and standard deviation, respectively. Values in square brackets indicate the 95% confidence interval for each correlation. **p < 0.01.

Table S4.

Means, standard deviations, and t-tests comparing group differences on the RCT outcome measures, excluding 15 children who used numbing spray and/or used distraction of a video playing during the venipuncture

Outcome variable	Mean (SD)	Significant difference between groups?	Cohen's *d* (95% CI)
1° Child fear	*Mind 2.35 (2.62)*	$p > .05$	-.11 (-.70, .47)
	Cont 2.05 (2.05)		
1° Child pain	*Mind 2.42 (2.43)*	$p > .05$	-.03 (-.61, .55)
	Cont 2.35 (2.40)		
2° Child rating of parent distress	*Mind 0.27 (0.72)*	$p < .001$***	0.67 (.06, 1.26)
	Cont 1.20 (1.96)		
2° Parent state distress	*Mind 1.31 (1.49)*	$p < .001$***	.56 (-.04, 1.15)
	Cont 2.55 (2.89)		
3° Parent rating of child fear	*Mind 3.15 (2.74)*	$p > .05$	.16 (-.43, .74)
	Cont 3.60 (2.89)		
3° Parent rating of child pain	*Mind 2.54 (2.08)*	$p > .05$	.29 (-.29, .88)
	Cont 3.20 (2.44)		

Note. (N=46 for all; n=26 in the mindfulness group, n=20 in the control group). ***$p < 0.001$. Cohen's d = 0.2, 0.5 and 0.8 represent a small, medium, and large effect size, respectively.
